# Drug-induced immune thrombocytopenic purpura secondary to sunitinib

**DOI:** 10.3747/co.v15i3.233

**Published:** 2008-06

**Authors:** M. Trinkaus, M. Trudeau, J. Callum

**Affiliations:** * Department of Medical Oncology, Sunnybrook Health Sciences Center, University of Toronto, Toronto, ON; † Department of Hematology, Sunnybrook Health Sciences Center, University of Toronto, Toronto, ON

**Keywords:** Breast cancer, thrombocytopenia, sunitinib

## Abstract

Sunitinib (Sutent: Pfizer, New York, NY, U.S.A.) is an oral multi-targeted tyrosine kinase inhibitor approved for use in various solid tumour malignancies. Many side effects secondary to sunitinib have been documented. In particular, sunitinib administration is known to result in thrombocytopenia, with the cause being attributed to myelosuppression. Here, we present the first case report to demonstrate immune-mediated thrombocytopenia secondary to sunitinib administration.

## 1. INTRODUCTION

Sunitinib (Sutent: Pfizer, New York, NY, U.S.A.) is an oral multi-targeted tyrosine kinase inhibitor, for which a role is being established as first- or second-line treatment in various malignancies. Sunitinib acts as an inhibitor of the vascular endothelial growth factor receptors 1, 2, and 3, and platelet-derived growth factor receptors a and b. It inhibits various tyrosine kinases such as stem-cell factor receptor Kit, colony stimulating factor 1, fetal liver tyrosine kinase receptor 3, and the glial cell line–derived neurotrophic factor receptor. In doing so, sunitinib has demonstrated reductions in tumour cell proliferation, increased apoptosis, reduced tissue invasion, and reduced angiogenesis 1,2.

To date, clinically approved uses for sunitinib include first-line treatment of metastatic renal-cell carcinoma and second-line treatment of gastrointestinal stromal tumours resistant to imatinib mesylate (Gleevec: Novartis Pharmaceuticals, St. Louis, MO, U.S.A.). However, sunitinib is in widespread use in clinical trials for both hematologic and solid-tumour malignancies. Toxicities reported with sunitinib use include hypertension, fatigue, skin toxicity (that is, hand–foot syndrome), diarrhea, vomiting, neutropenia, and thrombocytopenia 3,4. Here, we present the first reported case of drug-induced immune thrombocytopenic purpura secondary to sunitinib in a woman treated for metastatic breast cancer.

## 2. PRESENTATION

A 52-year-old woman with recently diagnosed hormone receptor–positive and human epidermal growth factor receptor 2–positive metastatic breast cancer was enrolled in a phase ii clinical trial in which she received sunitinib 50 mg daily and trastuzumab (Herceptin: Genentech, San Francisco, CA, U.S.A.) 8 mg/kg loading, and then 6 mg/kg every 3 weeks, as first-line treatment. The patient’s prior medical history was unremarkable for any autoimmune or hepatic disorders, hiv, or bleeding diatheses. She was a non-smoker and did not consume alcohol. Baseline complete blood count revealed hemoglobin 140 g/L, white blood cell count 9.4×10^9^/L, and platelet count 269×10^9^/L. Two weeks after starting the clinical trial, the patient was started on omeprazole for dyspepsia. She was not prescribed any other medications, and she did not subscribe to any non-conventional therapy.

Following 3 weeks of sunitinib treatment, the patient experienced a fall off a bicycle and noted excessive bruising over the areas of impact on her upper and lower extremities. She subsequently developed recurrent epistaxis refractory to conventional treatment measures, prompting her presentation to a community emergency department. At that time, the patient’s platelet count was 1×10^9^/L. Her bleeding stabilized after nasal packing and 5 units of transfused platelets.

### 2.1 Investigations

A complete blood count performed on the day following the platelet transfusion revealed hemoglobin 123 g/L, white blood cell count 4.0×10^9^/L, and platelet count 1×10^9^/L, suggesting a refractory response to platelet transfusion. Peripheral blood film reflected thrombocytopenia, with no evidence of schistocytosis, spherocytosis, or dysplasia. Baseline mean platelet volume had increased from 7.7 fL to 18 fL (normal range: 5–15 fL).

Other laboratory investigations yielded normal renal and liver function tests, a normal coagulation screen (international normalized ratio, prothrombin time, partial thromboplastin time), and a normal electrolyte profile. Physical examination was remarkable for oropharyngeal petechiae and diffuse ecchymoses, petechiae, and some purpuric lesions on the extremities. No splenomegaly was present.

The patient was admitted to hospital with a diagnosis of refractory thrombocytopenia. Notably, the last dose of trastuzumab had been administered the week before this patient’s documented thrombocytopenia, and sunitinib had been taken the day before.

### 2.2 Course of Treatment

Preliminary diagnosis was drug-induced immune thrombocytopenic purpura. The patient was treated with intravenous immunoglobulin (ivig) at 70 g (1 g/kg) over 2 days, and she also received tranexamic acid. Her platelet count rapidly improved to 101×10^9^/L (7 days after the ivig treatment) and returned to her normal baseline of 236×10^9^/L after 2 weeks.

Repeat imaging within 3 weeks of this patient’s last treatment with sunitinib and trastuzumab revealed growth of her visceral metastases. Trastuzumab was reintroduced at a lower weekly dose (2 mg/kg) with no drop in platelet count.

Normalization of this patient’s platelet count post ivig and post withholding of sunitinib is fully consistent with immune-induced thrombocytopenia secondary to sunitinib. She was thereafter started on alternative systemic treatment with good response and stability in her platelet count.

## 3. DISCUSSION

Immune-mediated thrombocytopenia is associated with various autoimmune disorders, hiv, hematologic malignancies, chronic infections, and medications. Drug-induced immune thrombocytopenia is thought to be a result of antibody production in the presence of a sensitizing medication, with the antibodies targeting epitopes on the platelet surface, subsequently leading to clearance of the antibody-coated platelets by the mononuclear phagocytic system 5.

Bleeding occurs in autoimmune thrombocytopenia not because antibody-coated platelets are dysfunctional, but rather because platelet numbers decline to at least 50×10^9^/L or lower 6. Drug-induced immune thrombocytopenia usually results in a drop in platelet count within 5–7 days of first medication exposure 5. Unlike primary or idiopathic thrombocytopenic pur-pura, drug-induced autoimmune thrombocytopenia usually resolves following withdrawal of the offending agent.

Autoimmune thrombocytopenia at time of diagnosis or during the course of breast cancer has been documented in case reports, suggesting the rarity of this phenomenon 7,8. The small number of cases of immune thrombocytopenia in conjunction with breast cancer adds to the difficulty of determining an actual pathophysiologic link between the two conditions. One small series of 10 breast cancer patients with immune thrombocytopenia suggested a mechanism of tumour-induced immunologic thrombocytopenia 8.

Sunitinib has been documented to result in thrombocytopenia, with the causation attributed mainly to myelosuppression. In a phase i dose-escalation trial involving various types of cancer, sunitinib at 50 mg daily caused thrombocytopenia in 2 of 9 patients 5. In a phase iii trial of metastatic renal cell cancer patients, Motzer *et al.* 3 reported some grade of thrombocytopenia in 65 of 375 patients randomized to sunitinib treatment, with 8 of the 65 (12%) having grade 3 thrombocytopenia.

One case of sunitinib-induced malignant hypertension causing thrombocytopenia secondary to micro-angiopathy has been reported 9.

Ours is the first documented case of a temporal association of sunitinib administration with a resultant immune-related thrombocytopenia. The possibility of an immunologic phenomenon to account for sunitinib-induced immune thrombocytopenia is reasonable, given its association with exacerbation of other autoimmune disorders such as hypothyroidism 10. The exact mechanism by which sunitinib induced an immune-mediated thrombocytopenia is unknown.

## 4. CONCLUSIONS

Our case raises the possibility that the new multi-targeted tyrosine kinase inhibitors such as sunitinib may have multiple side effects that have not yet been fully elucidated. In the context of thrombocytopenia following initiation of sunitinib, clinicians need to have a high index of suspicion for the potential of immune-mediated thrombocytopenia. In this regard, the medication can safely be discontinued.
